# Synthetic peptides as a novel approach for detecting antibodies against sand fly saliva

**DOI:** 10.1371/journal.pntd.0007078

**Published:** 2019-01-24

**Authors:** Michal Sima, Blanka Ferencova, Tapan Bhattacharyya, Michael A. Miles, Sergey V. Litvinov, Asrat Hailu, Gad Baneth, Petr Volf

**Affiliations:** 1 Department of Parasitology, Faculty of Science, Charles University, Prague, Czech Republic; 2 Faculty of Infectious and Tropical Diseases, London School of Hygiene and Tropical Medicine, London, United Kingdom; 3 Aptum Biologics Ltd, Southampton, United Kingdom; 4 Department of Microbiology, Immunology & Parasitology, Faculty of Medicine, Addis Ababa University, Addis Ababa, Ethiopia; 5 School of Veterinary Medicine, The Hebrew University of Jerusalem, Rehovot, Israel; Saudi Ministry of Health, SAUDI ARABIA

## Abstract

**Background:**

Hosts repeatedly bitten by sand flies develop antibodies against sand fly saliva and screening of these immunoglobulins can be employed to estimate the risk of *Leishmania* transmission, to indicate the feeding preferences of sand flies, or to evaluate the effectiveness of vector control campaigns. Previously, antibodies to sand fly saliva were detected using whole salivary gland homogenate (SGH) or recombinant proteins, both of which also have their disadvantages. This is the first study on sand flies where short peptides designed based on salivary antigens were successfully utilized for antibody screening.

**Methodology/Principal findings:**

Specific IgG was studied in hosts naturally exposed to *Phlebotomus orientalis*, the main vector of *Leishmania donovani* in East Africa. Four peptides were designed by the commercial program EpiQuest-B, based on the sequences of the two most promising salivary antigens, yellow-related protein and ParSP25-like protein. Short amino acid peptides were synthesised and modified for ELISA experiments. Specific anti-*P*. *orientalis* IgG was detected in sera of dogs, goats, and sheep from Ethiopia. The peptide OR24 P2 was shown to be suitable for antibody screening; it correlated positively with SGH and its specificity and sensitivity were comparable or even better than that of previously published recombinant proteins.

**Conclusions/Significance:**

OR24 P2, the peptide based on salivary antigen of *P*. *orientalis*, was shown to be a valuable tool for antibody screening of domestic animals naturally exposed to *P*. *orientalis*. We suggest the application of this promising methodology using species-specific short peptides to other sand fly-host combinations.

## Author summary

Previously, two types of antigens were used for detection of antibodies to sand flies: 1) salivary gland homogenate (SGH), which requires maintaining a sand fly colony and laborious work to obtain a sufficient amount of antigen or 2) recombinant proteins with the need to use cell expression and a complicated purification procedure. In contrast, synthetic peptides have never been studied for sand flies despite it being easier to produce them in sufficient quantities and purity. In this study, we screened specific antibodies to sand flies in domestic animals using synthetic peptides based on the two most antigenic salivary proteins of *Phlebotomus orientalis*. This sand fly is the main East African vector of *Leishmania donovani*, causative agent of visceral leishmaniasis, and we detected specific anti-*P*. *orientalis* IgG in naturally exposed dogs, goats, and sheep from Ethiopia. We showed that, in dogs and goats, the peptide named OR24 P2 is more suitable for antibody detection then the recombinant proteins. Therefore, we recommend this peptide to replace SGH in larger epidemiological studies for evaluation of the effectiveness of vector control programmes or to estimate the risk of *Leishmania* transmission.

## Introduction

The specific IgG antibody response against salivary proteins is induced in repeatedly exposed hosts after being bitten by the female sand fly (reviewed by Ribeiro and Francischetti [1] and Lestinova et al. [2]). In sand flies this antibody response is species-specific [3,4] and correlates with the biting intensity [5–8]. IgG values decrease after the hosts are protected against sand flies [9], therefore the detection of antibodies can be used for testing the efficacy of vector control campaigns [10,11].

Antibody detection with the whole salivary gland homogenate (SGH) as antigen is impractical in large epidemiological studies due to the possibility of crossreactivity with other insects [9], variability of saliva composition during sand fly aging [12,13], and the workload required to obtain sufficient quantity of the antigen. In the past decade, sand fly SGH was replaced by several antigenic recombinant proteins, expressed in bacterial or mammalian cells, and with various degrees of success (reviewed by Lestinova et al. [2]). In humans, successful detection of anti-sand fly IgG with recombinant proteins was described by Teixeira et al. [14] and by Souza et al. [15] for *Lutzomyia longipalpis* and by Marzouki et al. [16,17] and Mondragon-Shem et al. [18] for *Phlebotomus papatasi*. In domestic animals, using recombinant antigens, antibodies against sand fly saliva were detected in sera of dogs bitten by *L*. *longipalpis* or *P*. *perniciosus* [14,19,20] and in sera of dogs, sheep, and goats exposed to *P*. *orientalis* [21]. In wild animals these studies were performed with rabbits and hares bitten by *P*. *perniciosus* [22] and with foxes exposed to *L*. *longipalpis* [14].

However, production of recombinant proteins requires cell expression and a complicated purification procedure. Therefore, we focused on linear B-cell epitopes (synthetic peptides, representing short amino acid sections of the antigenic proteins), which can be produced in large amounts with high purity. This approach was previously applied to mosquitoes as well as to tsetse flies. In *Anopheles gambiae* the peptide designed based on the salivary protein gSG6 was validated in many field studies [23–26] and promising results were also achieved with peptide based on the salivary protein of *Aedes aegypti* and human serum samples [27]. In tsetse flies, peptides originating from saliva of *Glossina palpalis gambiensis* and *G*. *morsitans* specifically bound anti-tsetse fly antibodies in human and cattle sera, respectively [28–30].

In this study we applied, for the first time, this novel approach to sand flies and used short peptides to detect specific IgG response in domestic animals (dogs, goats, and sheep) naturally exposed to *P*. *orientalis*. Our main aim was to compare the peptides with previously described recombinant proteins [21] and to assess whether this methodology is also applicable to large scale surveillance.

## Materials and methods

### Ethical statement

BALB/c mice were maintained and handled in the animal facility of Charles University in accordance with institutional guidelines and the Czech legislation (Act No. 246/ 1992 coll. on Protection of Animals against Cruelty in present statutes at large), which complies with all relevant European Union and international guidelines for experimental animals. The experiments were approved by the Committee on the Ethics of Animal Experiments of the Charles University (Permit Number: MSMT-10270/2015-6) and were performed under the Certificate of Competency (Registration Number: CZ 02457) in accordance with the Examination Order approved by Central Commission for Animal Welfare of the Czech Republic. Sera of domestic animals were collected within the study by Rohousova et al. [31]. Their collection was approved by the Ethiopian National Research Ethics Review Committee (NRERC) under approval no. 3.10/3398/04.

### Host sera

Sera of domestic animals naturally exposed to *P*. *orientalis* in Ethiopia were obtained during the previous study by Rohousova et al. [31] and include 40 sheep, 94 goats, and 30 dogs. Sera from 10 sheep, 15 goats, and 10 dogs from non-exposed animals originating from the Czech Republic served as negative controls. More details of all the samples are provided by Rohousova et al. [31]. Twenty laboratory Balb/c mice were divided into four groups of five animals. Three groups were exposed at least ten-times to about 150 insectary-bred sand fly females (at two-week intervals) of either *P*. *orientalis*, *P*. *papatasi*, or *Sergentomyia schwetzi*; the fourth group was used as the non-exposed control.

### Sand flies and salivary gland dissection

The *Phlebotomus orientalis* colony originating from Ethiopia (for more details see Seblova et al. [32]) was reared under standard conditions as described by Volf and Volfova [33]. Salivary glands were dissected from 4–6 day old female sand flies in 20mM Tris buffer with 150mM NaCl and stored at -20°C. Before use, salivary glands were disrupted by freeze-thawing three times in liquid nitrogen.

### Peptide design and preparation

Peptides were designed from amino acid sequences based on the two most suitable recombinant proteins of *P*. *orientalis* (rPorSP24 and rPorSP65) as previously described [21]. Two peptides from each protein were selected in the software EpiQuest-B (Aptum Biologics Ltd., www.epiquest.co.uk). In EpiQuest-B, immunodominant parts of protein sequences were distinguished and their antigenicity indices were calculated based on three algorithms–the peptide immunogenicity, the probability of antibody-accessibility (exposure on the protein surface), and the uniqueness of protein sequence. The probability of peptide-antibody binding increases with the antigenicity index.

These four generated sequences (Table 1) were sent to a commercial laboratory (Genosphere Biotechnologies, France), where they were synthesised and conjugated with two molecules of polyethylene glycol, which acts as a spacer on ELISA plates and facilitates improved accessibility of antibodies. After the spacer, one molecule of biotin was added, which enabled avidin-biotin peptide binding to ELISA plates coated with diluted avidin. Peptides were diluted in sterile PBS at a concentration 1 mg/ml and stored in -80°C.

**Table 1:** Peptide description.

Peptide	Peptide sequence	Original protein ACCN
OR24 P1	GVGQVEYKGDEQKYPKGC	AGT96461 [34]
OR24 P2	GDSRQISCWNIQKPLNHGC	AGT96461 [34]
OR65 P1	GTPSTANQIDHYLNQIGC	AGT96466 [34]
OR65 P2	DRGVDGHNTDHEEYDYSC	AGT96466 [34]

Peptide names, sequences and accession numbers of the original proteins are indicated, with references.

### ELISAs

ELISA Clear Flat-Bottom Plates (3855: Thermo Fisher Scientific, USA) were coated with avidin (A9275: Sigma-Aldrich, UK) at a concentration 5 0μg/well, diluted in 20mM carbonate-bicarbonate buffer (pH 9.5) and incubated overnight at 4°C. Plates were washed three times with PBS-Tw (0.05% Tween 20), blocked with 6% blocking medium diluted in PBS (see S1 Table) for 2 hours at 37°C and then washed twice. Peptides diluted in 2% blocking medium in PBS-Tw were added to the wells at a concentration 5 μg/well and the plates incubated for one hour at 37°C. After washing three times, sera diluted in 2% blocking medium in PBS-Tw were incubated on the plates for one hour at 37°C. Plates were washed five times and secondary antibodies diluted in PBS-Tw were added and incubated for one hour at 37°C. Finally, plates were washed six times, the reaction developed with phosphate-citrate buffer (pH 5.5) in the dark for six minutes at room temperature and stopped with 10% sulfuric acid. The optical density was measured at 492 nm using the Infinite M200 microplate reader (Tecan, Switzerland). At each step, 100 μl of each solution per well was used and all serum samples were tested in duplicate.

When the salivary gland homogenate (SGH) was used as antigen, ELISA plates were coated with 0.2 gland/well [21]. The step with peptide incubation was replaced by incubation with 2% blocking medium in PBS-Tw and the rest of the protocol remained the same. Blocking media, sera and conjugate dilutions for individual host species are indicated in S1 Table.

### Statistical analysis

The non-parametric Spearman test was used to assess correlations between total anti-SGH and anti-peptide IgG levels using GraphPad Prism version 6 (GraphPad Software, Inc., San Diego, CA, USA). For evaluation of the possible crossreactivity with other sand fly species the non- parametric Wilcoxon Rank-Sum test in GraphPad Prism version 6 was used. Statistical significance was considered when the p-value was < 0.05. Cut-off values were calculated from the mean optical density of control sera plus 3 standard deviations. The optical density values of anti-SGH antibodies were used as the gold standard to validate peptides in ELISA tests using positive (PPV) and negative predictive values (NPV), sensitivity, and specificity.

## Results

### Peptide design

**Fig 1:**
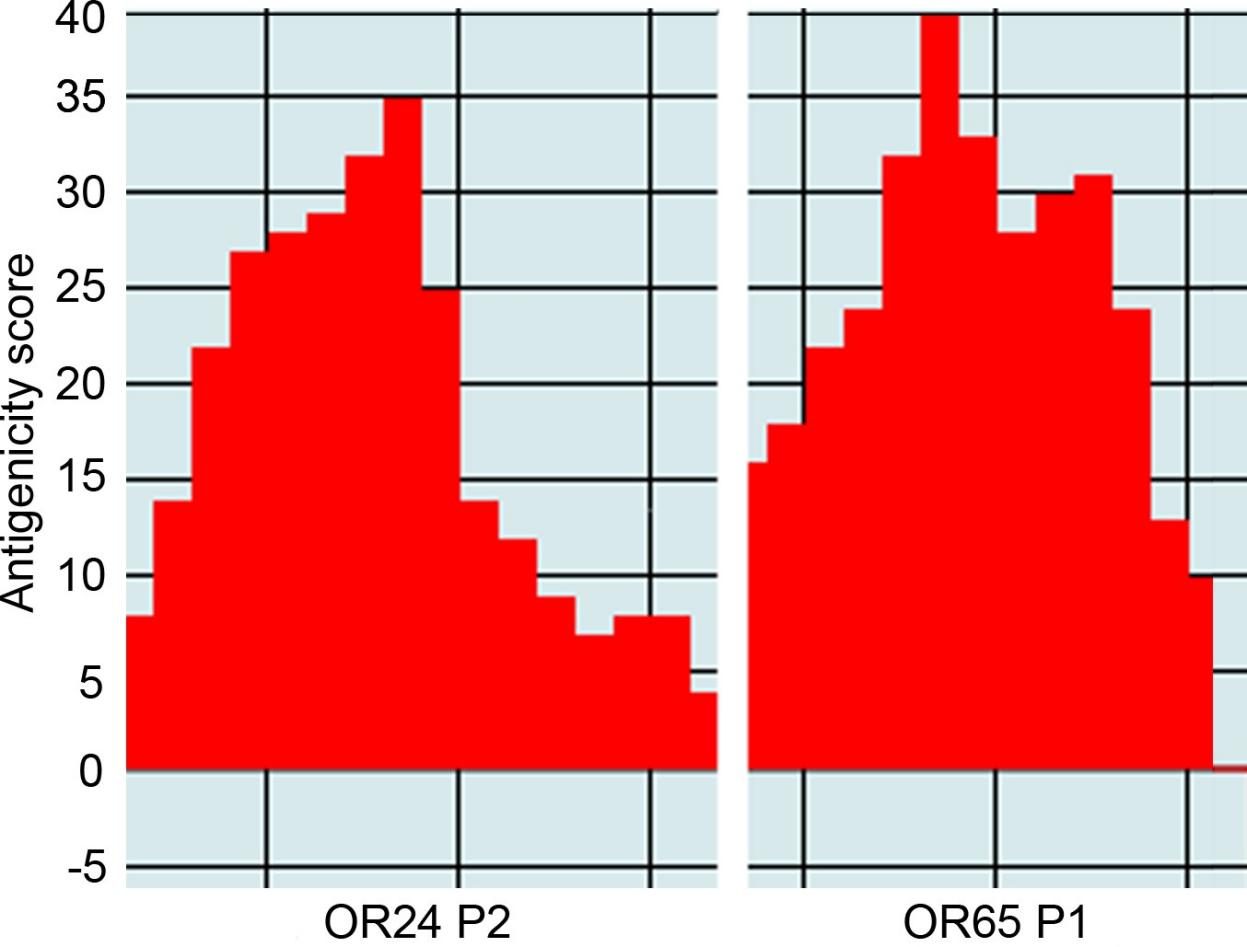
Computer-predicted antigenicity scores for two peptides. One peptide with the highest antigenicity score (calculated by EpiQuest-B) is displayed for each of the two proteins. Polymorphic sequences of OR24 P2 and OR65 P1 show regions of high antigenicity in red. Each red column represents one amino acid from the peptide sequence.

For designing the peptide sequences, two of the most antigenic proteins previously tested in recombinant form were used: rPorSP24 (yellow-related protein) and rPorSP65 (ParSP25-like protein). The antigenicity was calculated for both protein sequences in EpiQuest-B and two peptides with the higher antigenicity indices (Fig 1) were chosen from each protein: OR24 P1, OR24 P2, OR65 P1 and OR65 P2.

### Crossreactivity with sympatric sand fly species

**Fig 2:**
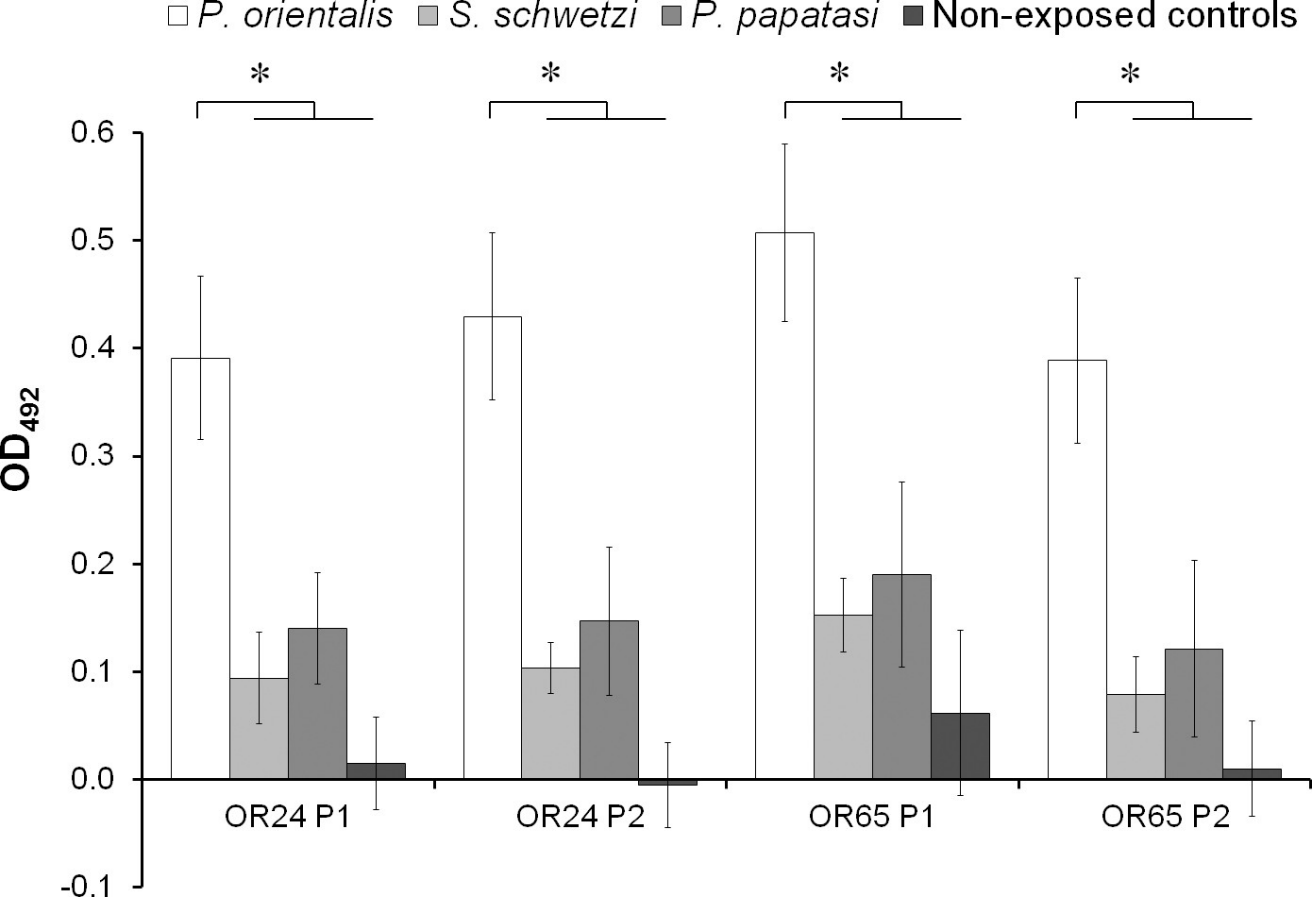
Specificity of peptides. Results from ELISA are presented as mean optical densities (with control values subtracted, from wells without serum) ± standard deviation of IgG antibody reaction, with four peptides (OR24 P1, OR24 P2, OR65 P1 and OR65 P2) and with sera from mice experimentally bitten by *P*. *orientalis*, *P*. *papatasi*, *S*. *schwetzi*, and non-exposed control mice. Five mice per sand fly colony and five non-exposed controls were used. Cut-off values were 0.144 for OR24 P1, 0.112 for OR24 P2, 0.292 for OR65 P1, and 0.143 for OR65 P2. Asterisks (*) indicate significant differences (p < 0.05, calculated with non-parametric Wilcoxon Rank-Sum Test) between IgG levels of mice bitten by *P*. *orientalis*, bitten by other sand fly species, and unbitten controls.

First, the synthetic peptides were tested by ELISA for possible crossreactivity with antibodies against salivary antigens of sympatric sand fly species (*P*. *papatasi* and *Sergentomyia schwetzi*) using sera of experimentally bitten Balb/c mice. Five mice were exposed to single sand fly species–either *P*. *orientalis*, *P*. *papatasi*, or *S*. *schwetzi*, and five mice served as non-exposed controls. Significant differences in OD values were detected with sera of mice bitten by *P*. *orientalis* compared to all the other three groups, as shown in Fig 2. No differences were observed in non-exposed controls and mice exposed to *P*. *papatasi* or *S*. *schwetzi* (Fig 2).

### ELISA experiments

The four aforementioned peptides were used as antigens in ELISA experiments to detect the specific anti-*P*. *orientalis* SGH antibodies from three animal species–dogs, goats, and sheep. Their antigenicities were compared with the whole SGH, and the statistical values calculated were cut-off, positivity, correlation coefficient, PPV, NPV, specificity, and sensitivity (Table 2).

**Table 2:** Evaluation of peptides in ELISA experiments.

HOST		SGH	OR24 P1	OR24 P2	OR65 P1	OR65 P2
**Dogs** (n = 30 +10)	Cut-off	0.417	0.481	0.587	0.607	0.883
	Positivity (%)	50.0	46.7	46.7	46.7	40.0
	Correlation	N.A.	0.764 ***	0.770 ***	0.774 ***	0.661 ***
	PPV		0.737	0.737	0.667	0.800
	NPV		0.909	0.909	0.889	0.800
	Specificity		0.667	0.667	0.533	0.800
	Sensitivity		0.933	0.933	0.933	0.800
**Goats** (n = 94 + 15)	Cut-off	0.499	0.546	0.656	1.111	0.532
	Positivity (%)	45.7	29.8	36.2	24.5	34.0
	Correlation	N.A.	0.631 ***	0.784 ***	0.550 ***	0.662 ***
	PPV		0.636	0.773	0.697	0.681
	NPV		0.700	0.820	0.672	0.766
	Specificity		0.686	0.804	0.804	0.706
	Sensitivity		0.651	0.791	0.535	0.744
**Sheep** (n = 40 + 10)	Cut-off	0.208	0.354	0.300	0.312	0.318
	Positivity (%)	10.0	2.5	7.5	2.5	5.0
	Correlation	N.A.	0.793 ***	0.426 ***	0.742 ***	0.571 ***
	PPV		1.000	0.333	0.333	1.000
	NPV		0.923	0.968	0.919	0.947
	Specificity		1.000	0.833	0.944	1.000
	Sensitivity		0.250	0.750	0.250	0.500

Sperman-Rank Correlation Matrix test for optical densities between sera tested against *P*. *orientalis* SGH and against each peptide, where the SGH was used as a golden standard. This table provides host species (with numbers of Ethiopian animals + non-exposed controls), cut-off values, positivity–the percentage of samples with OD values above cut-off, correlation coefficients, PPV, NPV, specificity, and sensitivity for four peptides and *P*. *orientalis* SGH. Asterisks (***) indicate significant correlation p < 0.001 and N.A. means not applicable.

OR24 P1 showed the closest cut-off value to SGH with canine sera, as well as high correlation coefficient (> 0.75), PPV, specificity (> 0.65) and very high NPV and sensitivity (> 0.9). With the goat sera, the correlation (0.6) was lower as were other statistical values (all between 0.6–0.7). With sheep sera, all statistical values were very high (> 0.9) except for the low sensitivity (0.25).

Peptide OR24 P2 had a high correlation coefficient with the SGH for dog and goat sera. It reached comparable PPV, NPV, specificity, and sensitivity as OR24 P1 for dogs and the highest PPV, NPV, specificity and sensitivity for goats (all above 0.75). Correlation and PPV (< 0.45) were low for sheep sera.

OR65 P1 showed similar statistical values as the previous two peptides for dogs but the specificity was slightly lower (0.5). With goat sera, there was a very high cut-off value and a low correlation coefficient (0.55). The second highest correlation (0.7) was achieved with this peptide and with sheep sera but the PPV and sensitivity (< 0.35) were low.

Despite high statistical values for OR65 P2 and canine sera (0.8), high cut-off and the lowest correlation coefficient (0.65) were observed with this host species. In contrast, OR65 P2 was the second best antigen for goats, with the lowest cut-off value, high correlation, PPV, NPV, specificity, and sensitivity (all above 0.65). Although high PPV, NPV, and specificity (> 0.9) were detected with sheep sera, this peptide achieved low correlation and NPV (< 0.6).

**Fig 3:**
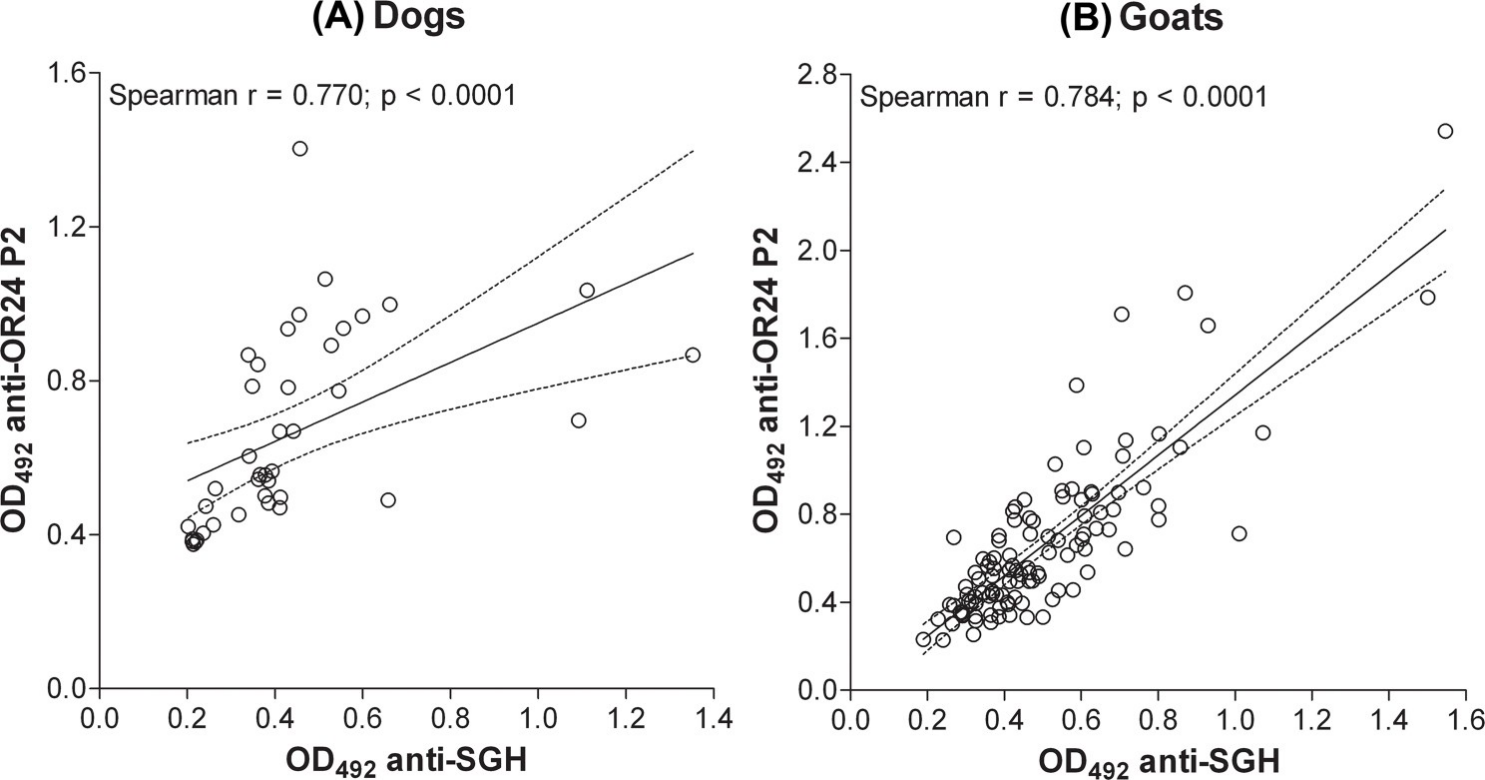
Correlation analyses between anti-*P*. *orientalis* IgG detected by SGH and by peptide OR24 P2 in ELISAs. For dogs (n = 40) and goats (n = 109) the peptide with the highest statistical values is displayed. Correlation coefficients and p-values from Spearman-Rank analysis are indicated.

The correlation analysis for the most promising peptide (OR24 P2) with dogs and goats is shown in Fig 3.

## Discussion

Previously, anti-sand fly IgG was detected by using SGH or recombinant proteins prepared based on the most antigenic proteins (reviewed by Lestinova et al. [2]). In this study, we focused on the novel approach of detecting IgG with short amino acid chain synthetic peptides. These peptides can be synthesised in large amounts with very high purity and without the need for cell expression of recombinants. We designed four peptides, two from each of the most promising *P*. *orientalis* salivary proteins: yellow-related protein (PorMSP24, ACCN: AGT96461) and ParSP25-like protein (PorMSP65, ACCN: AGT96466), and tested them with sera of domestic animals naturally exposed to *Phlebotomus orientalis*.

*Phlebotomus orientalis* is the main East African vector of *Leishmania donovani*–the causative agent of visceral leishmaniasis [35]. Previous studies revealed that salivary antigens of *P*. *orientalis* belong to several protein families, specifically yellow-related proteins, odorant-binding proteins, apyrases, antigen 5-related proteins and ParSP25-like proteins [34]. Recombinant yellow-related proteins and ParSP25-like proteins were used to replace *P*. *orientalis* SGH for antibody screening of domestic animals in Ethiopia [21].

Application of recombinant yellow-related proteins of *Lutzomyia longipalpis* was also described for canine, fox, and human sera [14,15], and of *P*. *perniciosus* for hare, rabbit, and canine sera [19,20,22]. However, so far, the recombinant ParSP25-like protein has not been used for other sand fly species except *P*. *orientalis* [21].

Peptides based on salivary antigens have not previously been used for studies on sand flies but they have been applied to detection of specific antibodies in hosts bitten by mosquitoes or tsetse flies. For mosquitoes, the peptides were first used for *Anopheles gambiae*, to study specific IgG responses among humans living in different foci of *Plasmodium falciparum* transmission [23,25,36], to correlate IgG levels with the risk of malaria transmission [26], and to monitor the effect of vector control campaigns [24]. Ndille et al. [27] used salivary peptide of *Aedes aegypti* to describe a positive correlation between specific IgG responses in humans with rainfall and mosquito seasonality. In tsetse flies, differences in anti-salivary peptide IgG titers were observed between two human populations with diverse abundance of *Glossina palpalis gambiensis* [28], and before and after vector control [29]. Somda et al. [30] suggested that the peptide based on salivary protein of *G*. *morsitans* was not suitable for IgG screening of domestic animals in areas with high tsetse fly abundance, because it was only recognized by sera of cattle with low exposure. Our study is the first, for sand flies, in which antigenicity and IgG detection are compared for peptides, whole SGH and recombinant proteins.

The peptides designed for *P*. *orientalis* are species-specific; no crossreactivity was observed with sera of mice exposed to the sympatric sand fly species *P*. *papatasi* and *Sergentomyia schwetzi* or with sera of non-exposed controls; there was similar high species specificity for the SGH and recombinant proteins of *P*. *orientalis* [21].

Previous work on dogs with recombinant proteins showed that the best protein-SGH performing recombinant (ParSP25-like protein) had very low specificity. This implied high probability of false positivity among non-exposed animals. Higher specificity was achieved with recombinant yellow-related protein [21]. Comparable correlation with recombinant yellow-related protein was detected with three peptides–OR24 P1, OR24 P2, and OR65 P1. The first two of these peptides also showed much higher specificity (0.7) than both of the recombinants.

With goats, low correlation was observed with both recombinant proteins [21]. In contrast, peptide OR24 P2 based on the sequence of yellow-related protein reached high correlation with SGH (0.8) as well as values > 0.75 for PPV, NPV, specificity, and sensitivity.

Results with sheep sera were difficult to interpret due to the very low positivity with SGH (10%). Similar positivity was found with all four peptides: even a relatively small change in the number of false positives or false negatives would significantly change calculation of PPV, NPV, specificity, or sensitivity. Although the correlation with two peptides (OR24 P1 and OR65 P1) was above 0.7, we therefore do not recommend these peptides for screening sheep sera. However, promising results for sheep sera have previously been achieved with both recombinant proteins [21].

In summary, we tested four short amino acid sequence peptides, designed based on two most antigenic *P*. *orientalis* salivary proteins, for detection of antibodies to sand fly saliva, in three species of domestic animals from Ethiopia. One of the peptides, OR24 P2, showed promising results with sera of dogs and goats. We therefore suggest that this peptide may replace SGH or recombinant proteins in surveillance for anti-*P*. *orientalis* IgG. As it was shown, synthetic peptides might work only for some host species. For future detection of human antibodies to sand fly saliva, we recommend comparison of the efficacy of recombinant proteins and synthetic peptides.

## Supporting information

S1 Table Dilutions in ELISA experiments. Host species, used blocking media and conjugates, sera and conjugate dilutions are indicated in this table.
(DOCX)
