# Supplementary material for: Synthetic peptides as a novel approach for detecting antibodies against sand fly saliva
Source: PLoS Negl Trop Dis. 2019 Jan 24;13(1):e0007078. doi: 10.1371/journal.pntd.0007078 (PMC6345433; doi:10.1371/journal.pntd.0007078)
Supplement: S1 Table — Host species, used blocking media and conjugates, sera and conjugate dilutions are indicated in this table. (DOCX) [file pntd.0007078.s001.docx]

| **HOST** | **Blocking medium** | **Sera dilution**  **(for SGH/**  **for peptides)** | **Conjugate** | **Conjugate dilution**  **(for SGH/**  **for peptides)** |
| --- | --- | --- | --- | --- |
| **Sheep** | Inactivated horse serum (16050-122:  Thermo Fisher Sci., USA) | 1: 50 | Rabbit anti-Sheep IgG (NB7195:  Novus Biol., USA) | 1: 10 000 / 1: 5 000 |
| **Goats** | Normal rabbit serum  (S-5000:  Vector Lab., USA) | 1: 50 / 1: 100 | Rabbit anti-Goat IgG (NB710-H:  Novus Biol., USA) | 1: 5 000 / 1: 7 000 |
| **Dogs** | Nonfat dry milk  (170-6404:  Bio-Rad, USA) | 1: 200 | Sheep anti-Dog IgG (A40-123P:  Bethyl Lab., USA) | 1: 3 000 / 1:5 000 |
| **Mice** | Nonfat dry milk  (170-6404:  Bio-Rad, USA) | 1: 50 | Goat anti-Mouse IgG (172-1011:  Bio-Rad, USA) | 1: 750 |
